# Mechanisms of stearoyl CoA desaturase inhibitor sensitivity and acquired resistance in cancer

**DOI:** 10.1126/sciadv.abd7459

**Published:** 2021-02-10

**Authors:** Nicole Oatman, Nupur Dasgupta, Priyanka Arora, Kwangmin Choi, Mruniya V. Gawali, Nishtha Gupta, Sreeja Parameswaran, Joseph Salomone, Julie A. Reisz, Sean Lawler, Frank Furnari, Cameron Brennan, Jianqiang Wu, Larry Sallans, Gary Gudelsky, Pankaj Desai, Brian Gebelein, Matthew T. Weirauch, Angelo D’Alessandro, Kakajan Komurov, Biplab Dasgupta

**Affiliations:** 1 Division of Oncology, Cincinnati Children’s Hospital Medical Center, Cincinnati, OH, USA.; 2 Human Genetics, Cincinnati Children’s Hospital Medical Center, Cincinnati, OH, USA.; 3 College of Pharmacy, University of Cincinnati, Cincinnati, OH, USA.; 4 Experimental Hematology and Cancer Biology, Cincinnati Children’s Hospital Medical Center, Cincinnati, OH, USA.; 5 Center for Autoimmune Genomics and Etiology, Cincinnati Children’s Hospital Medical Center, Cincinnati, OH, USA.; 6 Division of Developmental Biology, Cincinnati Children’s Hospital Medical Center, Cincinnati, OH, USA.; 7 Department of Biochemistry and Molecular Genetics, University of Colorado Anschutz Medical Campus, Aurora, CO, USA.; 8 Neurosurgery, Brigham and Women’s Hospital, Boston, MA, USA.; 9 Ludwig Institute of Cancer Research, University of California, San Diego, CA, USA.; 10 Memorial Sloan Kettering Cancer Center, New York, NY, USA.; 11 Department of Chemistry, University of Cincinnati, Cincinnati, OH, USA.; 12 University of Cincinnati College of Medicine, Cincinnati, OH, USA.; 13 Divisions of Biomedical Informatics and Developmental Biology, Cincinnati Children’s Hospital Medical Center, Cincinnati, OH, USA.

## Abstract

The lipogenic enzyme stearoyl CoA desaturase (SCD) plays a key role in tumor lipid metabolism and membrane architecture. SCD is often up-regulated and a therapeutic target in cancer. Here, we report the unexpected finding that median expression of SCD is low in glioblastoma relative to normal brain due to hypermethylation and unintentional monoallelic co-deletion with phosphatase and tensin homolog (PTEN) in a subset of patients. Cell lines from this subset expressed undetectable SCD, yet retained residual SCD enzymatic activity. Unexpectedly, these lines evolved to survive independent of SCD through unknown mechanisms. Cell lines that escaped such genetic and epigenetic alterations expressed higher levels of SCD and were highly dependent on SCD for survival. Last, we identify that SCD-dependent lines acquire resistance through a previously unknown FBJ murine osteosarcoma viral oncogene homolog B (FOSB)–mediated mechanism. Accordingly, FOSB inhibition blunted acquired resistance and extended survival of tumor-bearing mice treated with SCD inhibitor.

## Teaser

Genetic and epigenetic mechanisms determine SCD inhibitor sensitivity and acquired resistance through FOSB in cancer.

## INTRODUCTION

The degree of unsaturation and length of fatty acid chains in membrane lipids exert a profound influence on the fluidity of biological membranes—a disequilibrium in saturated to unsaturated fatty acid (UFA) ratio alters cell growth, differentiation, and response to external stimuli (*1*–*3*) and thus affects a range of pathologies including cancer. Overload of saturated fatty acids (SFAs) or cholesterol can cause membrane rigidity, cytotoxicity, and endoplasmic reticulum (ER) stress (*4*). The most abundant fatty acids in membrane phospholipids and glycolipids are the 16 and 18 carbon fatty acids. In a critical reaction dependent on cytochrome b5 and molecular oxygen, the ER-resident ∆^9^desaturase enzyme stearoyl CoA desaturase (SCD) catalyzes the desaturation of saturated fatty acyl-CoA substrates, such as stearoyl-CoA (C18) and, to a lesser extent, palmitoyl-CoA (C16), at the delta-9 position, to synthesize oleoyl-CoA (C18:1) and palmitoleyl-CoA (C16:1), respectively (*1*, *2*). UFAs such as oleate are crucial for membrane structure and function because they introduce a bend to generate a fluid (“liquid crystalline”) membrane (*5*, *6*). This biophysical property is essential for macromolecule transport, particularly in the ER, the major site of protein and lipid synthesis (*5*, *6*). Hence, by regulating cholesterol efflux, SCD modulates membrane domain structure (*7*). The amount of SCD in a cell is mainly regulated at the transcriptional level. Several transcription factors (TFs), hormones, and dietary elements regulate SCD expression suggesting a context-dependent fine regulation of the gene. These regulators include sterol regulatory element-binding protein 1 (SREBP1c), retinoid X receptor (RXR), liver X receptor (LXR), peroxisome proliferator-activated receptor alpha (PPARα), peroxisome proliferator-activated receptor gamma coactivator 1-alpha (PGC1α), insulin, growth hormone, androgens, ghrelin, and SFAs among others, while polyunsaturated fatty acids (PUFAs), cholesterol, glucagon, leptin, and triiodothyronine (T3) repress SCD expression (*8*–*10*). High SCD expression correlates with higher monounsaturated fatty acid (MUFA) to SFA ratio in membranes of highly motile cells such as macrophages (*11*), indicating a potential role of SCD in cell migration.

Mice express four *SCD* isoforms (SCD1 to SCD4). SCD1 null mice show improved insulin sensitivity, higher-energy metabolism, and resistance to diet-induced obesity (*12*, *13*). Humans polymorphic for rare SCD alleles show improved insulin sensitivity (*14*). Unlike mice, humans express only two paralogs—*SCD* and *SCD5* (*3*). Human SCD shares ~85% amino acid identity with mouse SCD1-4. In contrast, *SCD5*, which arose through genome duplication, shares limited homology with the rodent SCD’s and is unique to primates (*15*). SCD is ubiquitously expressed in human tissues with the highest level of expression in the adipose tissue, brain, and liver (*16*). In contrast, SCD5 has limited expression outside the brain, adrenal gland, and ovary. SCD5 expression in the brain, although highest among all tissues, is still substantially less than that of SCD (*16*). SCD mRNA levels strongly correlate with oleate levels in subregions of the adult human brain (*17*).

The physiological importance of SCD5 is not clearly understood in either normal physiology or pathology. Only SCD is up-regulated in several human cancers [(*18*, *19*) http://firebrowse.org/; https://oncomine.org/resource/login.html], and genetic disruption of only SCD but not SCD5 kills cancer cells (*20*–*22*). SCD overexpression in cancer cells alters membrane fatty acid composition. For example, relative to the normal human breast, membranes of the highly aggressive triple negative breast cancer cells are abundant in MUFA (*23*, *24*). While the essential PUFAs (linoleic and linolenic acids) must be obtained only through diet, MUFAs such as oleic acid can either be obtained through diet or synthesized de novo. However, dietary oleate cannot fully compensate for the lack of de novo SCD activity since both genetic and pharmacological inhibition of SCD in mice alters membrane lipid composition and are consequential in both normal physiology and pathology including cancer (*12*, *21*, *25*–*28*). Several mitogens turn on SCD expression implicating its role in cell division (*29*), a process that is enhanced in transformed cells. Oncogene-induced transformation augments SCD expression, MUFA synthesis, incorporation of newly synthesized MUFA to membrane phospholipids, and membrane fluidity (*21*, *30*). The high-lipogenic phenotype of many tumors also necessitates high-SCD expression because SFAs, like palmitate and stearate that are continuously produced in these tumors, inhibit the activity of acetyl coA carboxylase (ACC; the rate limiting FA synthesizing enzyme), and thus by simultaneously desaturating these SFAs, SCD relieves this inhibition on lipogenesis (*29*). Given the importance of SCD in cancer pathology, several SCD inhibitors are being developed for preclinical testing (*31*).

When chemotherapeutics are moved from preclinical testing to human studies, their success is limited by a multitude of factors including acquired resistance of tumor cells. Despite the potential of SCD inhibitors to reach the clinic, we know little about tumor cell resistance to such therapy. We initiated a study to interrogate the efficacy of SCD inhibition in glioblastoma (GBM), a disease with universal lethality, and to examine the mechanisms of resistance to SCD inhibitors. In this study, we report a number of unexpected and serendipitous findings including hemizygous passenger co-deletion of SCD with phosphatase and tensin homolog (PTEN) on chromosome 10, methylation of the trans-SCD allele in a subset of patients, intrinsic resistance of lines derived from this subset to SCD inhibitors despite retention of residual SCD activity, and discovery of a universal mechanism of SCD inhibitor acquired resistance in cancer. We propose that while SCD inhibitors have a therapeutic window, SCD expression and methylation status may serve as exclusion criteria in oncology clinical trials.

## RESULTS

### SCD expression may serve as a biomarker for SCD inhibitor sensitivity in cancer

**Fig. 1:**
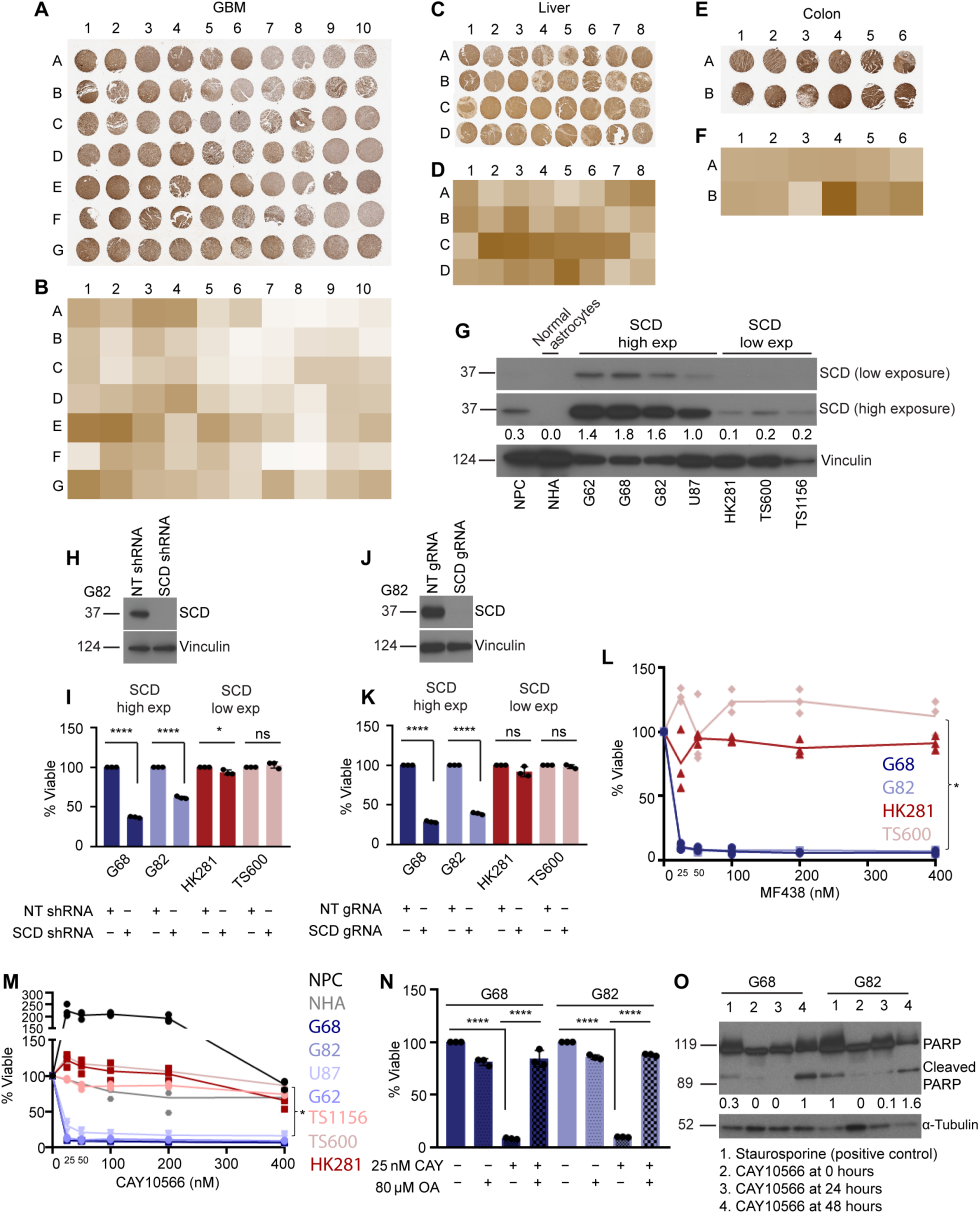
SCD expression determines sensitivity to SCD inhibition. (**A** to **F**) TMAs showing SCD protein expression and quantification in GBM (A and B), liver cancer (C and D), and colon cancer (E and F). (**G**) SCD protein in normal human NPCs, NHAs, and patient-derived GBM lines (G62, G68, G82, U87, HK281, TS600, and TS1156). Vinculin was used as loading control. Densitometry for SCD high exposure is shown. (**H** and **J**) Western blot (WB) showing SCD knockdown efficiency by shRNA (H) and gRNA (J) in G82 cells. NT, nontarget. Vinculin was used as loading control. (**I** and **K**) Viability of high-SCD (G68 and G82) and low-SCD (HK281 and TS600) GBM cells transduced with NT shRNA/gRNA, SCD shRNA (I), or SCD gRNA (K) at 72 hours (*n* = 3; ****P = <0.0001 and *P = 0.0205). ns, not significant. (**L** and **M**) Viability of high-SCD and low-SCD GBM cells in the presence or absence of the SCD inhibitors MF438 (L) or CAY10566 (M) at 72 hours (*n* = 3; *P < 0.0001). A linear regression model was used to obtain statistical significance. (**N**) Viability of high-SCD GBM cells (G68 and G82) in the presence or absence of 25 nM CAY10566 and supplemented with or without 80 μM bovine serum albumin–conjugated oleic acid (OA) at 72 hours (*n* = 3; ****P = <0.0001). (**O**) WB of high-SCD GBM cells (G68 and G82) showing cleaved PARP protein in staurosporine (positive control) or CAY10566-treated cells. Staurosporine was used for 3 hours. α-Tubulin was used as loading control. Densitometry analysis of bands is shown. The results shown as mean plus SEM. WB represents data from two or three independent experiments. Unprocessed blots are available in fig. S8.

Examining multiple published datasets, we observed that although median SCD expression is high in several human cancers relative to normal tissue counterparts (fig. S1, A to C and E), there are exceptions. For example, median SCD expression is low in GBM relative to normal brain (fig. S1, D and E). Immunohistochemistry (IHC) of tissue microarray (TMA) samples showed that SCD expression in individual patients within a cancer type such as liver and colon cancer was variable, but this variability was much greater in GBM (Fig. 1, A to F). We confirmed specificity of the SCD antibody by staining tissues reported in Human Protein Atlas and National Center for Biotechnology Information (NCBI) to express high (adipose tissue of human breast) or low (human bone marrow) levels of SCD (fig. S1, F and G). We were curious about the high variability of SCD in GBM and tested SCD protein expression in both serum-free patient-derived xenograft lines and commonly used serum-dependent cell lines. Protein analysis segregated GBM lines into two subsets—one that expressed high to moderate levels of SCD (henceforth high-SCD lines) and another where SCD was undetectable (henceforth low-SCD lines) (Fig. 1G) until blots were overexposed when the linearity of signal is often lost. SCD protein was also undetectable in cultured normal human astrocytes (NHAs), while low levels of SCD were observed in normal human neural progenitor cells (NPCs) in overexposed blots (Fig. 1G). Several SCD inhibitors have been patented for preclinical use against metabolic disorders and cancer. We tested whether SCD expression has any bearing on inhibitor sensitivity. While high-SCD GBM lines showed exquisite sensitivity to multiple *SCD* short hairpin RNA (shRNA), guide RNA (gRNA), and inhibitors, low-SCD GBM lines were refractory (Fig. 1, H to M, and fig. S1, H and I). Both NPCs and NHAs were also resistant (Fig. 1M). Exogenous oleic acid (the product of SCD) completely blunted the effect of the inhibitor confirming its specificity and precluding off-target effects (Fig. 1N). Consistent with other studies (*20*–*22*), SCD inhibition caused apoptosis in high-SCD GBM lines (Fig. 1O and fig. S1J). Because all of the SCD-high lines were sensitive and the SCD-low lines were resistant, our data demonstrate that at least in GBM and perhaps other human cancers, SCD expression could be a determining factor during patient selection for SCD inhibitor therapy.

### SCD inhibition impedes in vivo growth of high-SCD GBM lines

**Fig. 2:**
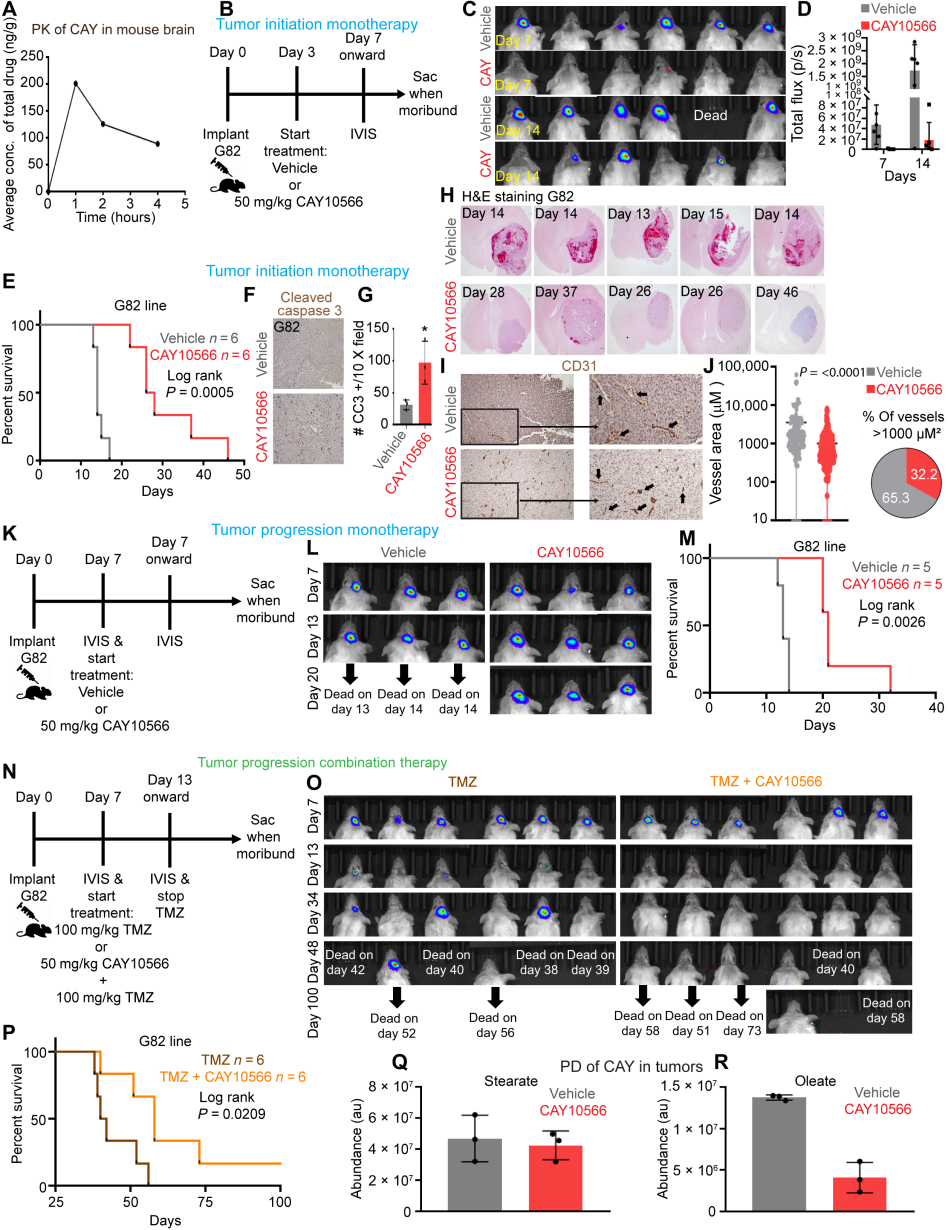
SCD inhibitor shows therapeutic efficacy in GBM. (**A**) Oral PK of CAY10566 (50 mg/kg) in NSG mouse brain tissue at indicated time points. (**B**) CAY10566 dosing schedule to monitor growth of high-SCD G82 tumors during the initial (lag) phase of tumor growth. (**C**) Luciferase imaging of mice to monitor tumor growth at the indicated days. (**D**) Luminescence quantification is shown as total flux (p/s). (**E**) Kaplan-Meier survival curve of mice transplanted with G82 cells. Mice were treated with vehicle or CAY10566. *n* = 6 per group. *P* = 0.0005. (**F** and **G**) IHC using cleaved caspase 3 antibody in vehicle- or CAY10566-treated mice. *P* = 0.02. (**H**) Hematoxylin and eosin (H&E) staining of tumors harvested at indicated days. (**I**) IHC using CD31 antibody in vehicle- or CAY10566-treated mice. (**J**) Quantification of tumor blood vessel area in inhibitor- or vehicle-treated mice. (**K**) CAY10566 dosing schedule to monitor growth of high-SCD G82 tumors during the exponential (log) phase of tumor growth. (**L**) Luciferase imaging to monitor tumor growth at the indicated days. (**M**) Kaplan-Meier survival curve of mice transplanted with G82 cells. Mice were treated with vehicle or CAY10566. *n* = 5 per group. *P* = 0.0026. (**N**) Dosing schedule of CAY10566 and TMZ to monitor growth of high-SCD G82 tumors during the exponential (log) phase of tumor growth. (**O**) Luciferase imaging to monitor tumor growth at the indicated days. (**P**) Kaplan-Meier survival curve of mice transplanted with G82 cells. Mice were treated with TMZ or TMZ + CAY10566. *n* = 6 per group. *P* = 0.0209. (**Q** and **R**) Pharmacodynamic (PD) studies showing the abundance of free stearate and oleate in tumors from vehicle- or CAY10566-treated mice (*n* = 3 vehicles and 3 CAY10566). au, arbitrary units.

Given the high efficacy and specificity of SCD inhibitors toward high-SCD lines in vitro, we wished to methodically test their therapeutic value in GBM. The blood-brain barrier (BBB) is sometimes breached in GBM; nevertheless, we performed pharmacokinetic (PK) studies to examine the BBB penetration of one SCD inhibitor, CAY10566. We examined CAY10566 enrichment in brain extracellular fluid (ECF) of Sprague-Dawley rats and in brain tissue of non-obese diabetic scid gamma (NSG) mice. In rats, the peak concentrations (*C*_max_) were 11,140.73 ng/g for plasma and 7.73 ng/g for brain ECF, a measure of unbound fraction of drug in the brain (fig. S2, A and B). Area under curve (AUC_0−*t*_) for rat plasma and ECF were 73,860.83 ng/ml per hour and 4731.77 ng/ml per hour, respectively. The time to peak (*T*_max_) was ~4 hours; half-life (*t*_1/2_) was 3.59 hours and 8.68 hours for plasma and brain ECF, respectively (fig. S2, A and B). In the mouse brain tissue, *C*_max_ (200.86 ng/g) was reached at 1 hour (Fig. 2A). We also tested whether SCD inhibitors would remain effective in vivo because, being an O_2_-dependent enzyme, SCD activity could be diminished in the highly hypoxic GBM microenvironment. To this end, we fed cells with U^13^C stearate and used liquid chromatography–mass spectrometry (LC-MS) to quantify U^13^C oleate synthesis under hypoxic conditions. We found that in hypoxic conditions that stabilized the hypoxia-inducible factor 1α (HIF1α) (fig. S2C), SCD retained >80% activity in up to 0.2% O_2_ and about 50% activity at 0.1% O_2_ (fig. S2D). This result minimized concerns about the efficacy of SCD inhibitors in hypoxic tumors. In three independent experiments, we tested whether SCD inhibition impedes tumor growth during the initial (lag) phase, exponential (log/progression) phase as a single agent (monotherapy), and during progression of advanced tumors in combination therapy with temozolomide (TMZ), the standard of care DNA alkylating agent for GBM. Despite a modest ECF to plasma ratio (6.4% of plasma), the SCD inhibitor showed significant therapeutic effects on tumor growth and survival in all three models. Initiating treatment in the lag phase (3 days after tumor cell implantation; Fig. 2B) significantly blocked tumor growth and improved survival in mice (Fig. 2, C to E). Mice treated with CAY10566 retained 80 to 90% body weight, which remained stable until the experiment end point (fig. S2E). No discernable difference in proliferative index was noticed in either early or late tumors of mice treated with vehicle or inhibitor (fig. S2, F and G). However, consistent with our in vitro results, inhibitor-treated tumors demonstrated considerably higher apoptosis than control tumors (Fig. 2, F and G).

Intracranial hemorrhage is a frequent complication in patients with primary brain tumor. Unexpectedly, in addition to reducing tumor burden, the SCD inhibitor blocked intracranial hemorrhage, suggesting a secondary effect on tumor vasculature (Fig. 2H). CD31 staining confirmed that control tumors contained large, abnormal vessels, whereas inhibitor-treated tumors contained small, normal vessels (Fig. 2, I and J). This result was corroborated by isolectin B4 staining that labels both endothelial cells and brain microglia/macrophages (fig. S2, H and I). Similar effects of the SCD inhibitor on tumor growth and survival were also observed in the inhibitor-sensitive U87 GBM line (fig. S2, J to N). We next investigated whether the SCD inhibitor can improve survival in mice with advanced tumors. Initiating treatment 7 days after tumor cell implantation when mice display fully established and advanced tumors, the SCD inhibitor was able to maintain stable disease and improved survival (Fig. 2, K to M, and fig. S2, O and P). Last, we tested whether SCD inhibition in combination with the GBM standard of care DNA alkylating agent TMZ improves survival in mice with advanced tumors compared to TMZ alone. TMZ alone caused significant tumor regression, although all mice relapsed, and 80% of mice died by day 50. In contrast, combination therapy allowed 80% of mice to survive past 50 days (Fig. 2, N to P). TMZ in combination with the SCD inhibitor reduced tumor burden and caused considerably more apoptosis relative to TMZ alone (fig. S2, Q and R). Last, to confirm that the SCD inhibitor hits target in tumors, we performed LC-MS of vehicle and inhibitor-treated tumors and quantitated stearate and oleate levels. This pharmacodynamic (PD) study showed that the SCD inhibitor significantly reduced oleate levels in tumors, without having any effect on stearate levels (Fig. 2, Q and R). Consistent with our in vitro results, SCD inhibitor–resistant HK281 tumors were refractory to the SCD inhibitor CAY10566, which failed to improve survival in mice (fig. S2S). Together, these results demonstrate that the SCD inhibitor CAY10566 penetrates the BBB, hits the target, reduces tumor burden, blocks hemorrhage, extends survival of mice with intracranial GBM as a single agent, and improves survival in combination with TMZ relative to TMZ alone.

### SCD undergoes bystander co-deletion with PTEN and epigenetic silencing in a subset of cancer

**Fig. 3:**
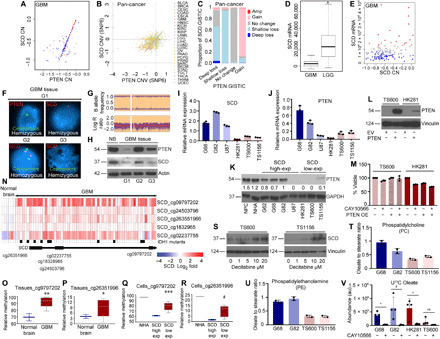
SCD undergoes co-deletion and epigenetic silencing in GBM. (**A**) CNV plot of *PTEN* and *SCD* in GBM patients (blue, low; red, high); *P* < 0.001. (**B** and **C**) SCD and PTEN copy number (CN) correlation in PAN cancer; *P* < 0.001. (**D**) Box plots showing SCD expression in GBM and LGG, (*n* = 548 GBM; 534 LGG); *P < 0.001. (**E**) Correlation of SCD CN and mRNA expression in GBM. (**F**) FISH analysis of *PTEN* and *SCD* in GBM tissues. (**G**) CNV array showing B allele frequency and log R ratio of SCD (see Materials and Methods). (**H**) WB showing PTEN and SCD in normal brain (NB) and GBM tissues. (**I** and **J**) SCD and PTEN mRNA expression in indicated GBM lines (*n* = 3). (**K**) WB and densitometry showing PTEN in indicated cells. (**L**) WB showing PTEN overexpression in low-SCD GBM cells; EV, empty vector. (**M**) Viability of cells in (L) with or without 25 nM CAY10566 (*n* = 3). (**N**) Heatmap and probe locations showing differential methylation ratio (log_2_) of SCD in GBM (*n* = 62) and normal brain (*n* = 6); black bars, IDH1 mutation. (**O** to **R**) Relative methylation of SCD in NB (*n* = 2) and GBM tissues (*n* = 8) using the indicated probes [(O) **P = 0.006; (P) *P = 0.09] and in NHA (*n* = 1) and GBM cells (*n* = 3 high-SCD and 5 low-SCD lines [(Q) ***P = 0.03; (R) #*P* = 0.046]. (**S**) WB showing SCD in low-SCD GBM cells treated with Decitabine. (**T** and **U**) Mass spectrometry showing oleate to stearate ratio in phospholipids of two high-SCD (G68 and G82) and two low-SCD (TS600 and TS1156) GBM cells (*n* = 2). (**V**) Oleate abundance in two high-SCD and two low-SCD GBM cells with or without 25 nM CAY10566 (*n* = 4; *P = 0.01, #*P* = 0.03, and +*P* = 0.02). Results shown as mean plus SEM. Unprocessed blots are in fig. S8.

Because SCD is overexpressed in several cancers (as shown in fig. S1), we were curious to understand why SCD expression is almost undetectable in a subset of GBM. *SCD* is located on chromosome 10 (10q24.31), about 12 Mb telomeric to the tumor suppressor (TS) gene *PTEN* (10q23.3). *PTEN* undergoes widespread functional inactivation in human cancer including GBM, melanoma, prostate adenocarcinoma (PRAD), and uterine corpus endothelial carcinoma (UCEC) through deletions, somatic mutations, and epigenetic silencing (hypermethylation of PTEN promoter) (*32*–*36*). Loss of heterozygosity (LOH) on chromosome 10 is the most frequent genetic alteration in GBM, occurring in up to 80% of cases (*37*, *38*). In the majority of primary GBM, chromosome 10 LOH involves loss of the entire chromosome 10 (*39*). We hypothesized that monoallelic loss of SCD is due to chromosome 10 LOH in GBM. To investigate whether low SCD expression is due in part to co-deletion with *PTEN*, we examined The Cancer Genome Atlas (TCGA) datasets. We found that *PTEN* copy number strongly correlated with *SCD* copy number and expression in GBM but not in other cancers such as colon and kidney cancer (Fig. 3A and fig. S3, A to C) and across all human cancers where PTEN deletion occurs. For example, we found SCD copy number loss in >90% of PTEN hemizygous loss (shallow) cancers, while no change in PTEN intact cancers (C); *P* < 0.001 (Fig. 3, B and C). Our analysis revealed that SCD undergoes copy number loss with PTEN in ~49% of melanoma, ~9% of PRAD, and ~9.6% of UCEC. Loss of SCD expression in PRAD was reported in a previous study (*40*). We also found significant correlation of PTEN copy number with that of several neighboring genes on chromosome 10q where *PTEN* is located (fig. S3, D to X). Copy number loss also strongly correlated with expression (fig. S3, D to X). Some of these monoallelic deletions include *FAS* (the cell death receptor), *GOT1*, and *GLUD1* (involved in glutamine metabolism), *PGAM1* (a glycolysis gene), *HIF1AN* (HIF1α inhibitor), and *MGMT* (a gene known to provide TMZ resistance in GBM through promoter methylation). We could not detect homozygous loss of these genes. Unlike GBM, *PTEN* deletions are rare in low-grade glioma (LGG). Accordingly, SCD expression was higher in LGG compared to GBM, indicating a positive correlation between *PTEN* deletion and *SCD* expression in brain tumors (Fig. 3D). Two additional pieces of information transpired from the TCGA GBM dataset: First, *SCD* copy number strongly correlated with SCD mRNA expression, and second, in a subset of GBM patients, particularly those with monoallelic SCD loss, SCD expression was nearly undetectable (Fig. 3E, blue dots between −1 and −0.4). Fluorescence in situ hybridization (FISH) using PTEN, SCD and chromosome 10 centromeric probes (Fig. 3F), and copy number variation (CNV) array (Fig. 3G) confirmed our analysis that indeed SCD undergoes hemizygous loss in PTEN deleted GBM. Western blots (WBs) from fresh GBM tissue lysates also showed a good correlation between PTEN and SCD protein levels (Fig. 3H).

Consistent with higher SCD protein levels in inhibitor-sensitive GBM lines (Fig. 1, G, L, and M), SCD mRNA was generally higher in these lines relative to inhibitor-resistant lines (Fig. 3I). Expectedly, the low-SCD resistant lines also showed lower PTEN mRNA (Fig. 3J) and protein (Fig. 3K). The U87 line that retains *PTEN* but with a homozygous splice site mutation leading to exon 3 deletion and no detectable PTEN protein expressed SCD (compare Figs. 1G and 3K) and was sensitive to SCD inhibitors (Fig. 1M). Because most low-SCD lines expressed very little PTEN protein (due to homo/hemizygous deletion, mutation, promoter methylation, or microRNA across all GBM subtypes), we tested whether PTEN loss played any role in SCD inhibitor resistance. To test this, we overexpressed PTEN in the SCD inhibitor–resistant lines and examined their sensitivity to SCD inhibitors. PTEN overexpressed cells retained resistance to SCD inhibition (Fig. 3, L and M), confirming that SCD inhibitor resistance is unrelated to PTEN expression. Human brain also expresses a paralog of *SCD* called *SCD5* that arose due to genome duplication (*41*). All known SCD inhibitors inhibit both SCD and SCD5; therefore, low-SCD lines that are resistant to SCD inhibitors and SCD shRNA/gRNA should also be resistant to SCD5 silencing. Nonetheless, we tested the role of SCD5 in these cells. We used human brain that expresses SCD5 (positive control) and human bone marrow that does not express SCD5 (negative control) (*16*) to optimize two SCD5 primers (fig. S4A). Using these primers, we found that overall, SCD5 mRNA levels were lower than SCD in all lines, but some low-SCD lines showed higher SCD5 mRNA relative to high-SCD lines (fig. S4B). However, SCD5 silencing (fig. S4C) had no effect on the viability of any cell line (fig. S4D), indicating that SCD5 inhibition is inconsequential in GBM. We also used two commercially available SCD5 antibodies that failed to distinguish between SCD5 proficient and deficient cells and, therefore, were deemed unreliable for our studies.

To verify the universality and functional consequence of PTEN-SCD co-deletion events in cancers outside the central nervous system, we turned to melanoma since PTEN is deleted in ~57% and ~38% of B-Raf proto-oncogene, serine/threonine kinase (BRAF) mutant and BRAF wild-type (WT) melanoma, respectively. Consistent with GBM, SCD copy number strongly correlated with that of PTEN in melanoma (fig. S4E), and SCD protein expression was also highly variable in melanoma TMA (fig. S4, F and G). Akin to GBM lines, melanoma lines also segregated into SCD inhibitor–sensitive or SCD inhibitor–resistant subgroups (fig. S4H). The sensitive subgroup is described in the literature as PTEN WT (both alleles intact, although silenced by mutation/methylation in some lines), while with resistant subgroup is annotated as PTEN hemizygous null. Analogous to GBM, the resistant lines expressed little or no SCD protein (fig. S4I). Similar to GBM, low and variable expression of SCD5 mRNA was detected in both SCD inhibitor–sensitive and SCD inhibitor–resistant melanoma lines (fig. S4J).

Because SCD is only hemizygously deleted, yet its expression was very low in SCD inhibitor–resistant lines, we questioned whether aberrant epigenetic silencing through hypermethylation of DNA played a role in the repression of the second SCD allele. Widespread cancer-specific differential methylation regions (CDMRs) of promoters, shores, shelves, and enhancers are found in all cancers including GBM (*42*–*44*). Methylation in these regions are generally associated with transcriptional repression. From the GSE36278 GBM methylome database (*45*), we chose to test several methylation-specific primers. Of these, cg24503796 (CpG island) and cg26351966 (N_Shore) were SCD promoter associated, cg09797202 was enhancer associated, and cg18328965 and cs02237755 were in CpG island and S-Shore, respectively. Cg24503796 and cg2237755 were also CDMRs. GSE36278 methylome data revealed differential methylation of the *SCD* locus in GBM relative to normal brain (Fig. 3N). Because the Isocitrate dehydrogenase 1 (IDH1) mutation often corresponds to GBM CpG island hypermethylator phenotype (G-CIMP), we cross-checked IDH1 mutation status in the GSE36278 cohort. IDH1 mutation was present only in 10 of 62 GBM patients (Fig. 3N), and we found no correlation between SCD methylation and IDH1 mutation. In addition, sequencing of three high-SCD and three low-SCD lines showed no IDH1 R132H mutation, suggesting that SCD methylation and IDH1 mutation are unlikely to be correlated. Pyrosequencing of normal human brain and GBM tissue using multiple methylation-specific probes indicated variable *SCD* methylation in GBM but with considerably higher median values relative to normal brain (Fig. 3, O and P, and fig. S4, K and L). Consistent with these results, low-SCD GBM and melanoma lines demonstrated significantly higher *SCD* methylation than high-SCD lines (Fig. 3, Q and R, and fig. S4, M and N), indicating that hypermethylation of the trans-SCD allele could be a reason for the nearly undetectable SCD expression in low-SCD lines. NHAs that did not show SCD protein (Fig. 1G) also demonstrated high-*SCD* methylation (Fig. 3, Q and R, and fig. S4M). Last, we tested whether treatment with the demethylating agent Decitabine could increase SCD expression in the low-SCD lines. Decitabine treatment for 24 hours increased SCD expression in these (TS600 and TS1156) lines (fig. 3S), indicating hypermethylation as the most plausible cause for low SCD expression in these cells.

### SCD inhibitor–resistant low-SCD lines retain residual SCD activity

SCD generates two monounsaturated fatty acyl-CoAs (MUFA), oleoyl-CoA, and palmitoleyl-CoA from stearoyl-CoA and palmitoyl-CoA, respectively. Because our GBM stem cell lines grow in medium without serum (a source of oleate and palmitoleate), we expected that low-SCD lines produce no or minimal MUFA. To identify the fatty acids associated with membrane phospholipids in these cells, we performed mass spectrometry–based lipidomics. Expectedly, oleate to stearate and palmitoleate to palmitate ratios in phosphatidylcholine (PC) and phosphatidylethanolamine (PE) were reduced in low-SCD lines (Fig. 3, T and U, and fig. S4, O and P). Long-chain MUFAs that are synthesized by C2 elongation of oleoyl-CoA were also reduced in low-SCD cells (fig. S4, Q and R). However, our data also revealed that the low-SCD lines do produce substantial amounts of MUFA, which is unexpected given the extremely low levels of SCD protein in these cells. This suggests high catalytic efficiency of the residual Δ^9^ desaturase or the presence of an unidentified alternative pathway of MUFA synthesis in these lines. Several long-chain PUFAs that are synthesized from the two diet/media-derived essential fatty acids—linoleate and linolenate—were up-regulated in low-SCD cells (fig. S4, S and T), presumably in response to lower MUFA levels. To test whether residual Δ^9^ desaturase activity is at least partially responsible for MUFA synthesis in low-SCD lines, we measured SCD activity. We labeled both high-SCD and low-SCD lines with U^13^C stearate in the presence or absence of SCD inhibitor for 24 hours and performed ultra-high performance liquid chromatography–MS (UHPLC-MS) analysis. Despite monoallelic deletion, methylation, very low SCD expression and resistance to SCD inhibitors, low-SCD lines retained residual Δ^9^ desaturase activity (Fig. 3V). Collectively, the above data demonstrate that while *SCD* undergoes epigenetic silencing and monoallelic co-deletion in a subset of *PTEN*-deleted cancer leading to significant loss of expression, residual SCD activity is present in these cells but inessential for their growth and viability.

### SCD inhibitor–sensitive lines acquire resistance by overexpressing SCD

**Fig. 4:**
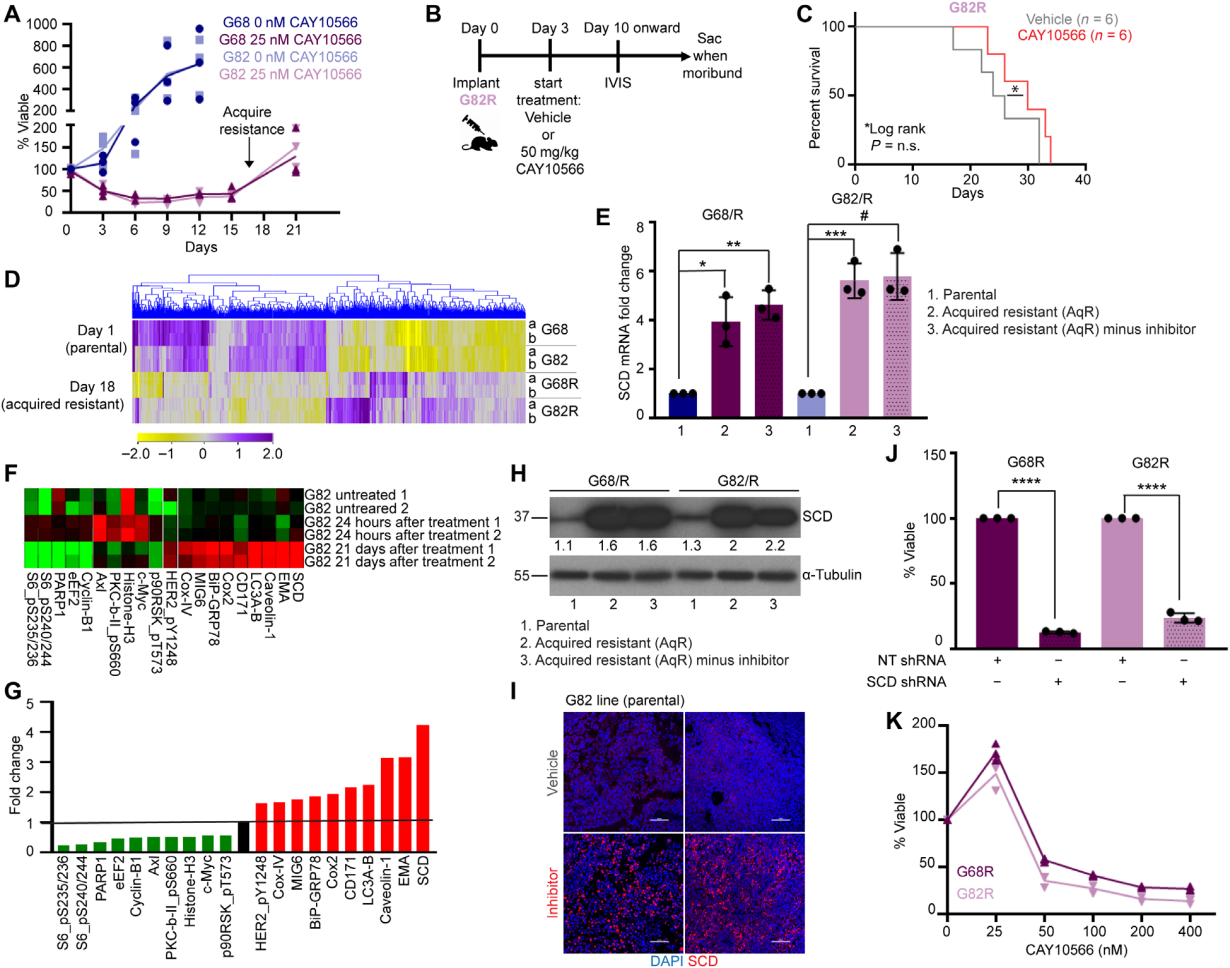
SCD inhibitor–sensitive lines acquire resistance by overexpressing SCD. (**A**) Viability of high-SCD GBM cells (G68 and G82) in the presence or absence of CAY10566 at the indicated days (*n* = 3). (**B** and **C**) Dosing schedule and Kaplan-Meier survival curve of mice transplanted with G82 AqR (G82R) cells. Mice were treated with vehicle or CAY10566. *n* = 6 per group. *P* = ns. (**D**) Heatmap and hierarchical clustering of the DEGs in AqR (G68R and G82R) and parental cells (G68 and G82). Duplicate samples were processed (yellow, low; purple, high expression). (**E**) qRT-PCR showing fold change in SCD mRNA expression in AqR lines (G68R and G82R) relative to parental lines (G68 and G82), in the presence or absence of CAY10566 for 7 days (*n* = 3; *P = 0.0005, **P = 0.0069, ***P = 0.001, and #*P* = 0.0004). (**F** and **G**) Heatmap and quantification of RPPA of G82 cells treated with vehicle or 25 nM CAY10566 for 24 hours and 21 days. Green, low; red, high. One and two are duplicate samples. (**H**) WB showing SCD expression in AqR (G68R and G82R) and parental lines (G68 and G82) in the presence or absence of CAY10566 for 7 days. Densitometry analysis of bands is shown. (**I**) IHC of tumor tissue using SCD antibody in vehicle- and CAY10566-treated mice. Nuclei were stained with 4′,6-diamidino-2-phenylindole (DAPI). Scale bars, 100 μm. (**J** and **K**) Viability of AqR cells expressing NT or SCD shRNA (*n* = 3; ****P = <0.0001) (J) and in the presence of CAY10566 (*n* = 3) (K). The results shown as mean plus SEM. WB represent data from two or three independent experiments. Unprocessed blots are available in fig. S8.

Acquisition of drug resistance is common in cancer and occurs through various mechanisms. We observed that continuous exposure of inhibitor-sensitive GBM and melanoma lines to the SCD inhibitor CAY10566 gave rise to drug-resistant populations around 3 weeks (Fig. 4A and fig. S5A). To verify whether the cells that acquired resistance to CAY10566 in vitro are truly resistant to the inhibitor in vivo, we implanted the acquired resistant (AqR) G82R (R, resistant) line into the cortex of NSG mice and treated them with CAY10566 or vehicle following the schedule shown in Fig. 4B. Growth kinetics of G82R tumors was slower than parental G82 tumors (compare Fig. 2D with Fig. 4C). Akin to our in vitro results, CAY10566 had no effect on the survival of mice implanted with G82R cells (Fig. 4C). To understand the mechanism/s of resistance, we performed next-generation sequencing (NGS)/RNA sequencing (RNA-seq) and functional proteomics [reverse phase protein array (RPPA)] in parental and AqR GBM lines. Analysis of RNA-seq data showed more than 600 differentially expressed genes (DEGs) (twofold up or down-regulated) including *SCD* in AqR cells relative to parental cells (Fig. 4D and table S1). Quantitative reverse transcription polymerase chain reaction (qRT-PCR) confirmed that *SCD* mRNA was highly up-regulated (four- to sixfold) in AqR cells. Furthermore, removal of the inhibitor for 7 days did not reduce SCD expression indicating a durable change in *SCD* regulation during acquisition of resistance (Fig. 4E). Unbiased RPPA of parental G82 and AqR G82R cells confirmed that among the hundreds of proteins with altered expression in G82R cells, SCD was the most up-regulated protein (>4-fold) in G82R cells compared to G82 cells (Fig. 4, F and G). WB analysis confirmed that SCD was indeed expressed at very high levels in GBM and melanoma AqR cells relative to parental cells (Fig. 4H and fig. S5B). Consistent with our in vitro data, inhibitor-induced SCD expression was also high in intracranial tumors (Fig. 4I). SCD protein also showed higher stability in AqR GBM cells (fig. S5, C to F), but this could not account for the robust SCD up-regulation in the AqR cells.

Before investigating the cause and consequence of SCD up-regulation in AqR cells, we wanted to confirm whether other genes in our RPPA and RNA-seq datasets that were up-regulated in AqR GBM cells contributed to SCD inhibitor resistance. To this end, we performed a barcoded pooled shRNA library screen of the top 38 genes up-regulated in AqR GBM cells. NGS and deconvolution were carried out after exposing AqR cells to shRNAs (two to nine independent shRNA against each gene in the pool). Representation of shRNA species was calculated to determine percent dropout (under representation, genes more important for viability) (fig. S5G). From this screen, the chemokine CCL2 emerged as one of the top hits. However, a CCL2-neutralizing antibody, despite significantly reducing viability, did not preferentially kill AqR cells relative to parental cells (fig. S5H). AqR GBM cells also showed up-regulation of LC3A-B and Beclin that regulate autophagy and GRP78 (Bip) that regulates ER stress and HER2 (human epidermal growth factor receptor 2) phosphorylation (Fig. 4, F and G). Because autophagy and controlled ER stress are adaptive mechanisms for cancer cell survival during stress, we tested whether inhibition of autophagy or overdriving ER stress preferentially sensitizes AqR cells. Neither Chloroquine (autophagy inhibitor) nor Eeyarestatin (ER stress inducer) showed preferential toxicity toward AqR cells, while the HER2 inhibitor Lapatinib had no effect on either parental or AqR cells (fig. S5, I to K). This indicates that these pathways are neither causal nor contributing but coevolved during acquisition of SCD inhibitor resistance.

We next tested whether SCD acquired any mutations in AqR cells. Sequencing showed no mutations in the coding region, including the eight conserved histidine residues that are critical for SCD catalytic activity (*1*). We argued that if drug-induced SCD up-regulation evolved to skew the stoichiometry of drug-inhibitor interaction and confer resistance to 25 nM SCD inhibitor [median inhibitory concentration (IC_50_) of parental cells is ~12.5 nM], then the AqR cells should respond to genetic suppression of SCD or higher dose of inhibitor. AqR GBM cells were sensitive to *SCD* shRNA (Fig. 4J) and higher doses of SCD inhibitor (IC_50_, ~50 nM) (Fig. 4K). These results suggest that AqR cells continue to rely on SCD for viability and growth. To investigate the mechanism/s of SCD overexpression, we first looked into SCD transcriptional regulation. *LXR* and *PPAR*α are among the several TFs that regulate *SCD* transcription in a stimulus-dependent manner (*8*, *46*). The PPARs can activate LXRα that, in turn, can activate *SCD* transcription through SREBP1c. Both PPAR antagonist GW9662 and LXR antagonist GSK2033 reduced SCD levels in parental cells but failed to do so in AqR GBM cells (fig. S5, L to O). Moreover, these known TFs for SCD were in fact down-regulated in AqR cells relative to parental cells (fig. S5P). Last, pyrosequencing showed no significant changes in SCD methylation in the AqR GBM lines compared to parental lines. Together, these results suggest that inhibitor-induced SCD overexpression is driven by transcription and is regulated by previously unknown mechanisms in AqR cells.

### SCD overexpression alters lipid profile of AqR cells

**Fig. 5:**
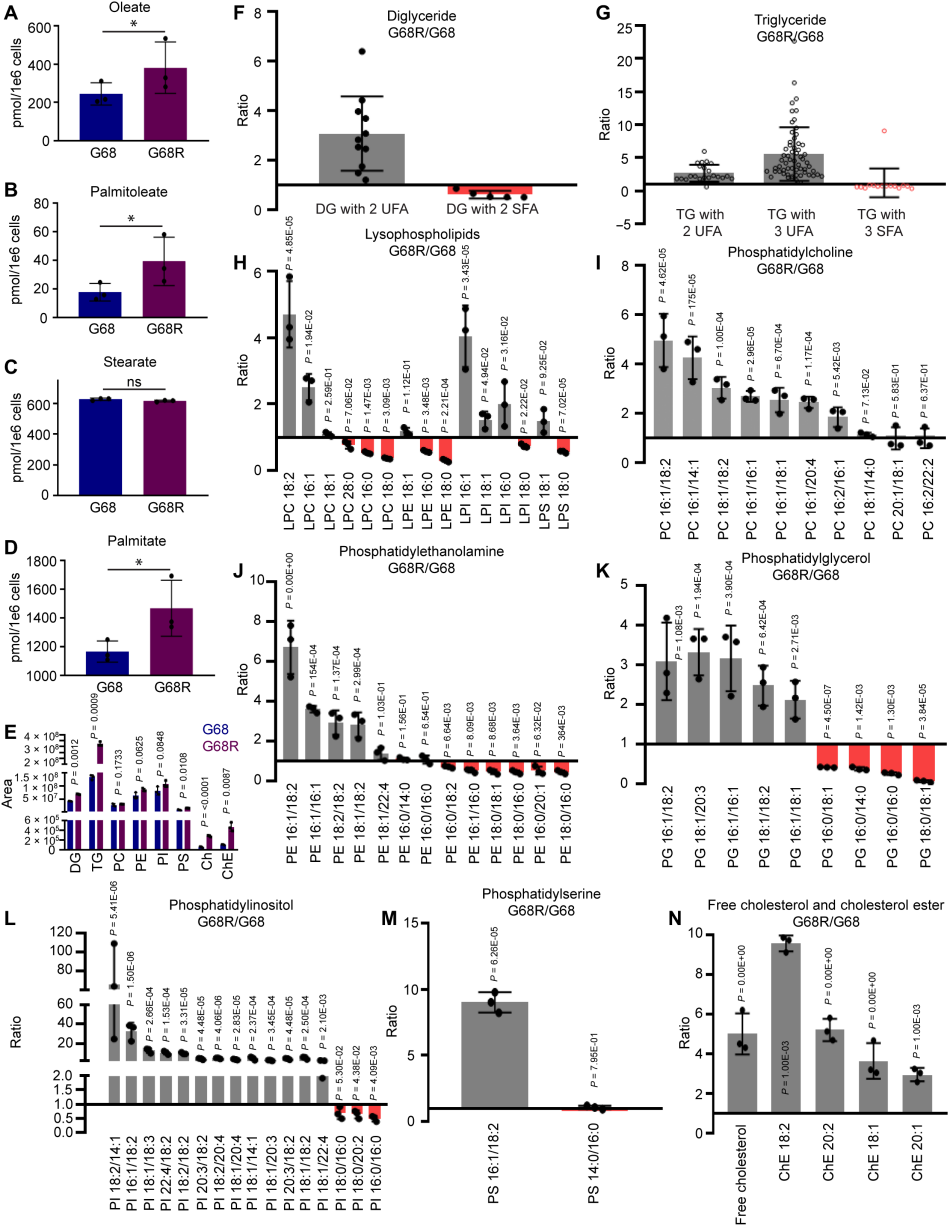
SCD inhibitor AqR cells undergo lipogenic reprogramming. (**A** to **D**) Untargeted lipidomic analysis showing oleate, palmitoleate, stearate, and palmitate levels in AqR (G68R) and parental (G68) cells (*n* = 3; *P = 0.05, 0.04, and 0.03, respectively). (**E**) Lipid levels in parental (G68) compared to AqR (G68R) cells. (PI, phosphatidylinositol; PS, phosphatidylserine; Ch, Cholesterol; ChE, Cholesteryl-ester) (*n* = 3). (**F** and **G**) DG or TG species expressed as ratio of G68R/G68. Each dot represents a DG or TG species with a combination of fatty acids different from the others. Gray, up-regulated; red, down-regulated. (**H** to **M**) Phospholipids (gray, up-regulated; red, down-regulated) in AqR (G68R) compared to parental (G68) cells expressed as ratio of G68R/G68. Fatty acids associated with each phospholipid species are shown in *X* axis. (**N**) Free cholesterol and cholesteryl-esters up-regulated in AqR (G68R) compared to parental (G68) cells expressed as ratio of G68R/G68. Fatty acids associated with cholesteryl-ester species are shown in *X* axis. Triplicate samples of G68 and G68R were analyzed.

SCD controls overall fatty acid biosynthetic rate and fatty acid composition of cellular lipids (*13*, *29*, *47*–*49*). Because inhibitor-induced SCD expression was several folds higher in AqR cells, we were curious to understand whether SCD up-regulation altered the lipogenic program of these cells. To this end, we performed untargeted lipidomic analyses in parental and AqR GBM cells. Levels of both oleate and palmitoleate were expectedly increased in AqR cells (Fig. 5, A and B). Total stearate levels were unchanged, but palmitate levels were high in AqR cells (Fig. 5, C and D), consistent with enhanced fatty acid synthesis in cells with high MUFA synthesis. Total levels of diglyceride (DG), triglyceride (TG), free cholesterol, and cholesteryl-esters were significantly higher in AqR cells relative to parental cells (Fig. 5E). Phospholipids including PC, PE, phosphatidylinositol, and phosphatidylserine also tended to be higher but did not reach statistical significance (Fig. 5E). Consistent with higher oleate levels, long-chain MUFAs that are synthesized from oleate were also high in AqR cells. Accordingly, DG and TG with two or more UFAs were overrepresented, whereas those with two or more SFAs were underrepresented in AqR cells (Fig. 5, F and G, and fig. S6, A and B). Similarly, in each class of phospholipid, lipids with UFA were overrepresented, while those with SFA were underrepresented (Fig. 5, H to M, and fig. S6, C to H). Free cholesterol and cholesteryl-ester levels were also higher in AqR cells (Fig. 5N and fig. S6I) presumably to offset high degree of unsaturation in membranes. Our data demonstrate inhibitor-induced SCD overexpression in cancer cells causes massive reprograming of the lipogenic pathway during acquisition of resistance to SCD inhibitors.

### Global changes in chromatin landscape and FOSB-mediated SCD overexpression in acquired-resistant cells

**Fig. 6:**
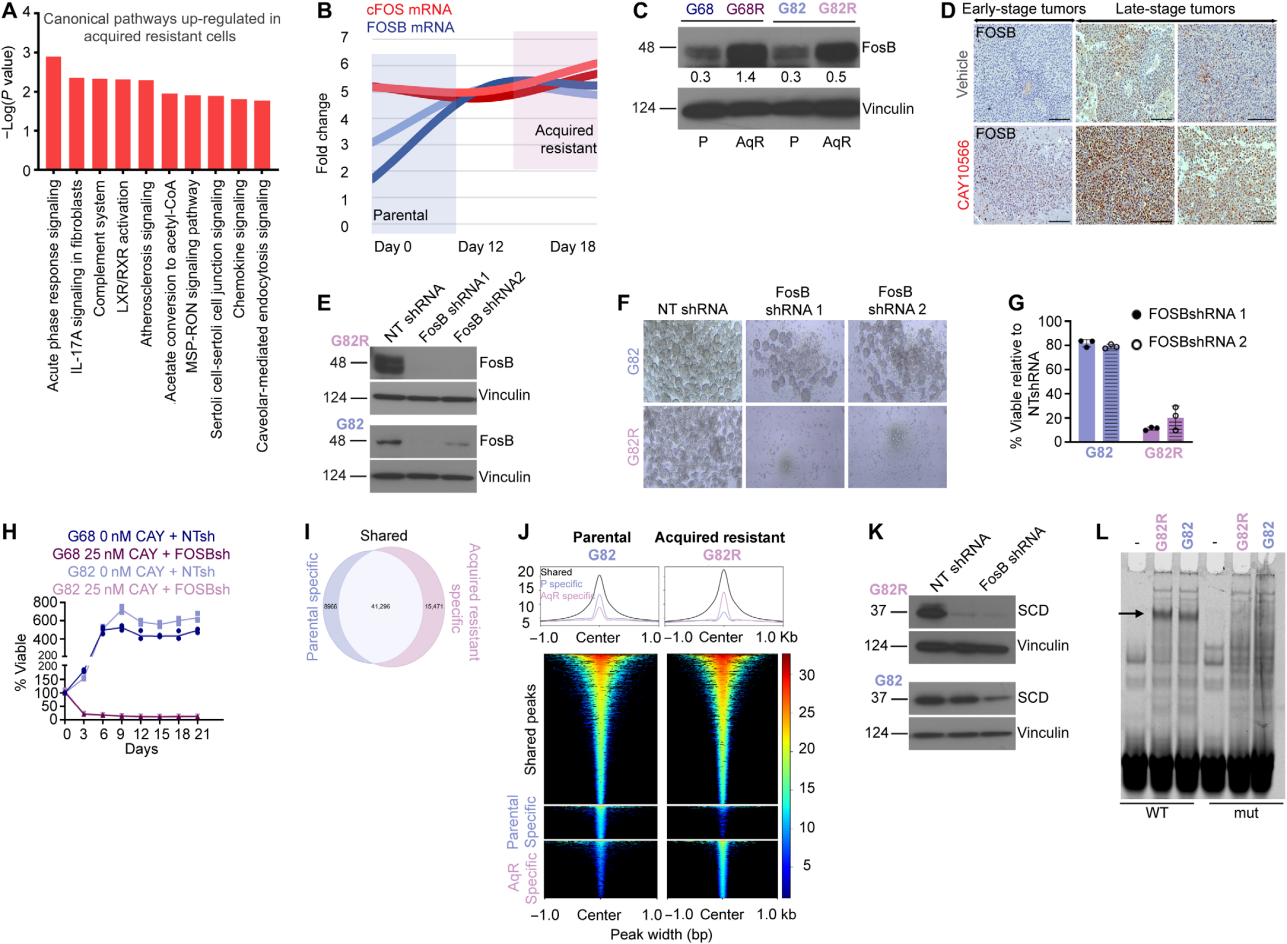
Chromatin reorganization and FOSB-driven SCD inhibitor acquired resistance in cancer. (**A**) Ingenuity pathway analysis of AqR and parental cells (see Materials and Methods). (**B**) FOSB and cFOS mRNA levels in parental (G68 and G82) and AqR (G68R and G82R) cells at the indicated days. (**C**) WB showing FOSB levels in parental and AqR cells. Densitometry analysis of bands is shown. (**D**) IHC using FOSB antibody in early or late stage intracranial tumors of mice treated with vehicle or CAY10566. Scale bars, 200 μm. (**E**) WB showing FOSB knockdown in G82R and G82 cells. NT, nontarget. (**F** and **G**) Bright-field images and viability of parental (G82) or AqR (G82R) cells treated with NT or FOSB shRNA, (*n* = 3). (**H**) Viability of parental GBM cells (G68 and G82) in the presence or absence of CAY10566 and FOSB shRNA at the indicated days (*n* = 3). (**I**) Venn diagram showing the number of ATAC-seq peaks in parental and AqR cells (see Materials and Methods). (**J**) Global changes in chromatin accessibility during the parental to AqR transition. Colors indicate the normalized number of sequencing reads. Average density plots are shown at the top. (**K**) WB showing SCD in NT or FOSB shRNA transduced G82R and G82 cells. (**L**) EMSA using nuclear extracts from parental (G82) and AqR (G82R) cells. WT or FOSB site mutated (mut) DNA probes from a predicted FOSB binding site in the SCD locus (green highlight) (see fig. S7B); (-) denotes no extract. The arrow indicates loss of binding when putative FOSB site is mutated. WB represents data from two or three experiments. Vinculin was used as loading control. Unprocessed blots are in fig. S8.

Because none of the known TFs appeared to regulate SCD in AqR cells, we performed Ingenuity Pathway Analysis (IPA) of our RNA-seq dataset to better understand the mechanism of drug-induced SCD overexpression. The top pathway identified by IPA was the acute phase response signaling regulated by the TF FOS (Fig. 6A and table S2). We found that while the FBJ osteosarcoma oncogene (cFOS) expression was similar in both parental and AqR GBM cells, FBJ murine osteosarcoma viral oncogene homolog B (FOSB) expression gradually increased during acquisition of resistance (Fig. 6B). Accordingly, FOSB protein levels were also significantly higher in AqR GBM and melanoma cells relative to parental cells, indicating a conserved mechanism of resistance across diverse tumors (Fig. 6C, and fig. S5Q). Consistent with our in vitro results, tumors in SCD inhibitor–treated mice, especially in later stages when tumors presumably start acquiring resistance showed robust up-regulation of FOSB compared to vehicle-treated mice (Fig. 6D). Collating our in vitro and in vivo data, this suggested that drug-induced FOSB induction is a potential mechanism of acquired resistance to SCD inhibitors in cancer. Two independent FOSB silencing RNAs that depleted FOSB protein levels (Fig. 6E) killed ~80% of AqR cells while having little effect on the parental cells (Fig. 6, E to G). Continuous exposure of inhibitor-sensitive parental GBM lines to the SCD inhibitor CAY10566 in the presence of FOSBshRNA did not give rise to resistant populations (Fig. 6H), indicating the requirement of FOSB for acquisition of AqR phenotype in these cells.

To examine FOSB binding sites and potential changes in chromatin landscape in AqR cells, we performed ATAC-seq (assay for transposase-accessible chromatin using sequencing). These ATAC-seq experiments revealed massive reprogramming of the chromatin landscape during transition from parental (sensitive) to acquired resistance. In total, we detected 60,338 ATAC-seq peaks in the parental and acquired resistance cells. Among these, 41,296 peaks were shared between parental and AqR cells, 8966 peaks were stronger in parental cells (parental specific), and 15,471 stronger in AqR cells (AqR specific) (Fig. 6, I and J). TF motif enrichment analysis revealed a strong presence for activator protein 1 (AP-1) site recognized by FOS, FOSB, and other basic leucine zipper domain (bZIP) proteins in both parental and AqR cells (fig. S7A and table S3). Thus, although AP-1 sites are highly present in both parental and AqR cells, the transition between the two states potentially resulted in repositioning of TFs such as FOSB across the genome, due to the large number of newly closed and open chromatin regions in the acquired resistance state (Fig. 6, I and J) and the high prevalence of AP-1 sites within these regions. In addition to AP-1, CTCF motifs are strongly enriched in AqR-specific peaks (and shared peaks) but not in parental-specific peaks (fig. S7A), suggesting that the newly opened chromatin regions in AqR cells are likely capable of forming looping interactions (*50*) with the promoters of new AqR-specific target genes. The *SCD* locus, in particular, contains parental-specific, AqR-specific, and shared chromatin accessibility regions, with most of these regions containing chromatin immunoprecipitation sequencing (ChIP-seq) peaks for FOS family members in a variety of cellular contexts (fig. S7B). We asked whether FOSB controlled SCD transcription and inhibitor-induced overexpression specifically in AqR cells. FOSB silencing abolished SCD expression in AqR GBM cells but not in parental GBM cells (Fig. 6K). TF motif analysis using our ATAC-seq data revealed a strong enrichment of potential FOSB binding sites in SCD regulatory regions. We therefore performed (ChIP) experiments. Two “ChIP-grade” FOSB antibodies failed to immunoprecipitate any chromatin, questioning the veracity of these reagents and precluding us from proceeding further. Because no suitable ChIP-grade FOSB antibodies were available to examine binding of endogenous FOSB to SCD regulatory regions, we performed electrophoretic mobility shift assays (EMSAs). Using FOSB motifs from build 2.0 of the CIS-BP database (*51*), we predicted a potential high-affinity FOSB binding site in the SCD locus. EMSA analysis of nuclear extracts showed specific binding to a WT DNA probe containing this region that was lost when the FOSB site was mutated, suggesting that FOSB can bind to the SCD locus in these cells (Fig. 6L). While our results are consistent with FOSB binding to the proposed site, we cannot eliminate the possibility that another protein binds to the FOSB binding site in the EMSAs. FOSB binding was observed in both parental and AqR cells. Perhaps SCD transcription is complex and disparate in parental and AqR cells, requiring a combinatorial transcriptional control by TFs such as SREBP1c, LXR, PPARα, and PGC1α in parental cells, with FOSB primarily driving SCD in AqR cells. It is worth recalling that SREBP1c, LXR, PPARα, and PGC1α were down-regulated in AqR cells compared to parental cells, and PPAR and LXR antagonists reduced SCD levels only in parental cells but not in AqR GBM cells (as shown in fig. S5, L to P).

### FosB silencing in parental GBM improves survival in mice treated with SCD inhibitor

**Fig. 7:**
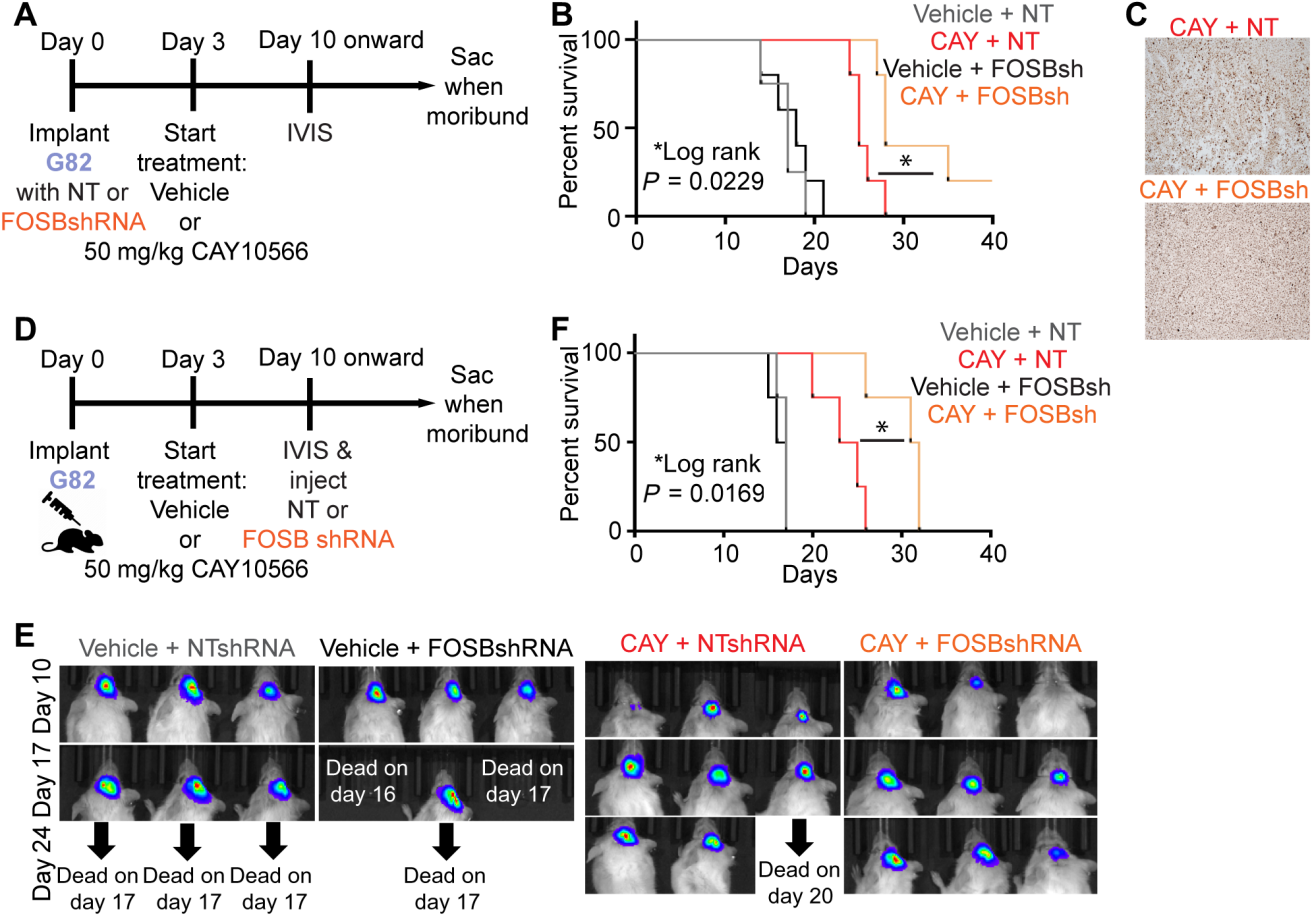
FOSB silencing in parental GBM improves survival in mice treated with SCD inhibitor. (**A**) Dosing schedule for high-SCD parental G82 cells transduced with NT or FOSBshRNA before implantation and treated with vehicle or CAY10566. (**B**) Kaplan-Meier survival curve of mice in the above experiment. *n* = 5 per group. *P* = 0.0229. (**C**) IHC of FOSB in intracranial NT or FOSBshRNA expressing tumors of mice treated with CAY10566. (**D**) Dosing schedule for high-SCD parental G82 cell implantation and treatment with vehicle, FOSB shRNA alone, CAY10566 alone, or in combination with FOSB shRNA. (**E**) Luciferase imaging to monitor tumor growth at the indicated days. (**F**) Kaplan-Meier survival curve of mice transplanted with G82 cells. Mice were treated with vehicle or CAY10566 and injected with NT shRNA or FOSB shRNA. *n* = 4 per group. *P* = 0.0169.

Next, we tested whether knocking down FOSB in parental cells before implanting them in mice would impede development of acquired resistance in vivo and improve survival in the SCD inhibitor–treated group (Fig. 7A). We observed that compared to CAY10566 treatment alone, CAY10566 treatment in combination with FOSBshRNA improved survival of mice implanted with the FOSB-silenced G82 parental line (Fig. 7B). We note here that these GBM cells are resistant to a host of antibiotics and hence we could not select them for FOSB shRNA expression. IHC for FOSB showed higher positivity in CAY10566 + NTshRNA–treated mice compared to CAY10566 + FOSBshRNA–treated mice (Fig. 7C) confirming FOSB knockdown. However, FOSB reactivity was still present in FOSB-silenced tumors suggesting the presence of nontransduced cells. The animals in the CAY10566 + FOSBshRNA group that died early showed much higher FOSB staining than the animals that survived longer, suggesting that incomplete FOSB silencing (or potential reactivation) may play a role in the variability in animal survival that we observed in this experiment. Next, to test whether reducing FOSB levels in advanced SCD inhibitor–treated tumors (when they presumably start acquiring resistance to the inhibitor) exerted any effect on tumor growth and survival, we performed an additional experiment. Despite technical challenges, we injected FOSB shRNA or nontarget shRNA into the tumor through the same burr hole that was used to inject parental G82 GBM cells 10 days after tumor cell implantation (Fig. 7D). FOSB shRNA alone did not decelerate tumor growth or improve survival (Fig. 7, E and F), confirming that FOSB has no significant role in tumor growth of parental cells. However, in SCD inhibitor–treated mice, where late-stage tumors overexpress SCD (see Fig. 4G), and presumably acquire resistance in vivo through FOSB overdrive (see Fig. 6D), FOSB shRNA slowed tumor growth and improved survival of mice (Fig. 7, E and F). Note that the effect of FOSB shRNA is partial, likely due to limited spread of lentivirus inside the tumor bed. Collectively, these results indicate that global changes in chromatin landscape, gene expression, and signaling occur during the evolution of SCD inhibitor resistance in cancer cells. FOSB-driven acute phase response signaling and FOSB-mediated SCD transcription and perhaps FOSB/SCD-independent mechanisms are involved in SCD inhibitor acquired resistance in cancer.

## DISCUSSION

The importance of SCD in membrane function and consequent up-regulation in cancer has been reported in several studies (*23*, *30*). SCD was reported to be necessary during both early states and the progression of lung tumorigenesis (*48*). Therefore, we were intrigued to find that in addition to monoallelic deletion, the SCD locus also undergoes unexpected hypermethylation and silencing in a subset of cancer cells. SCD was found to be one of the several hypermethylated genes in Glioma-CpG island methylator phenotype (G-CIMP) characteristic of proneural tumors (*44*). Driver alterations such as loss of TSs or oncogene amplification are critical for tumorigenesis, and as expected, they have been at the helm of investigation. Bystander alterations that arise either randomly in the genome or are accompanied with drivers are largely understudied (*52*–*54*). The TS *PTEN* is frequently deleted in GBM, sometimes as part of the 10q loss or chromosome 10 monosomy (*34*). Analyzing TCGA datasets, we found that several genes around *PTEN* are hemizygously co-deleted in GBM and other *PTEN* deleted cancers. These include the cell death receptor *FAS*, *MGMT* (that provides TMZ resistance in GBM through promoter methylation), *FGFR2*, glutamine metabolism genes such as *GOT1* and *GLUD1*, the multidrug resistance transporter *ABCC2*, and the glycolytic enzyme *PGAM1* among many others. SCD deletion and hypermethylation in PTEN-deleted cancers, however paradoxical, are inadvertent events in the cancer genome. It has been reported that collateral deletion of tumor promoting genes in fact cause hypomethylation and overexpression of the second allele as a compensatory mechanism (*55*). Therefore, hypermethylation and silencing of SCD, perhaps occurring along with epigenetic silencing of PTEN, is unexpected.

A limitation of our current findings, which is also constrained by the scope of the study, is that we do not fully understand the mechanisms of oleate synthesis and survival of the original SCD inhibitor–resistant (low-SCD) lines. MUFA synthesis from precursors was reduced in low-SCD lines, as expected. However, despite SCD deletion and methylation, how low-SCD lines synthesize a substantial amount of MUFA remains unknown. Why they synthesize MUFA when it is inessential for their growth and survival are intriguing questions. Although these cells retain ^Δ^9 desaturase activity, we are not certain whether all MUFA synthesized in these cells are products of SCD alone or also of a yet unknown activity that is insensitive to SCD inhibitors. This unknown activity may allow these cells to survive in the presence of SCD inhibitors. Alternatively, these cells could use back-up SCD-independent lipogenic pathways to survive. A recent study showed the presence of a SCD-independent lipid desaturation pathway in some lung and liver cancer cell lines (*56*). We are uncertain at this point whether this pathway is active in the SCD inhibitor–resistant GBM and melanoma lines. The significance of the evolutionary necessity of SCD/oleic acid–independent alternative pathways is unclear and warrants investigation in the future.

Despite modest BBB penetration, the SCD inhibitor CAY10566 was remarkably efficient in reducing oleate levels and blocking intracranial growth of tumors. BBB is often breached in GBM; therefore, the therapeutic concentration of the drug inside tumors could be higher. Consistent with our in vitro findings, SCD inhibition caused apoptosis without any appreciable change in Ki67 index, indicating that SCD may not be required during GBM cell cycle per se. We showed that SCD retains significant enzymatic activity even in highly hypoxic conditions—a result that should encourage SCD inhibitor therapeutics in other solid tumors. In addition to delaying tumor growth, the inhibitor had an unexpected beneficial effect on tumor vasculature, secondary to its effect on tumor cells. GBM is a highly vascular tumor; however, abnormally large blood vessels and malfunctioning vasculature often cause intratumoral hemorrhage and complications in patients (*57*). The SCD inhibitor almost completely blocked intratumoral bleeding and seemed to normalize blood vessel size. The notable effect of the inhibitor on tumor vasculature has potential therapeutic value because normalization of tumor vasculature may allow enhanced delivery of other anticancer drugs to synergize with SCD inhibition. CAY10566 and TMZ combination improved survival relative to either drug alone. We expect that an alternative drug scheduling where TMZ is delivered in multiple cycles may yield even better results.

Drug resistance is the underlying principle for cancer cell survival, tumor recurrence, and therapeutic failure (*58*). As expected, both GBM and melanoma cells acquired resistance to the SCD inhibitor. The consistency in the time to acquire resistance and the molecular mechanism that provided resistance in the two anatomically distinct tumor types was notable. The SCD inhibitor induced a durable reorganization of chromatin that affected thousands of loci some of which closed while others reopened in the AqR cells. Of the many changes that affected gene expression, SCD itself was found to be highly up-regulated in AqR cells—an effect noticeably consistent between GBM and melanoma cells. In multiple cancer models, modulation of SCD expression was associated with changes not only in MUFA levels, but biosynthesis of fatty acids, triacylglycerol, cholesteryl esters and phospholipids synthesis were also markedly affected (*30*, *48*, *49*), indicating that SCD controls the overall process of lipogenesis in cancer (*59*). Consistent with these studies, SCD up-regulation in AqR GBM cells increased MUFA levels and augmented palmitate, phospholipid, TG, and cholesterol synthesis.

Our finding that a single TF FOSB controls the transcriptional overdrive of SCD in AqR cells in two unrelated cancers is notable. The AP-1 TFs, consisting of *JUN*, *FOS*, and others have been implicated in drug resistance in other cancers (*60*). We observed that while the levels of cFOS remained stable, FOSB levels increased significantly in AqR GBM and melanoma cells. Four pieces of evidence suggest that FOSB up-regulation is a key mechanism of acquired resistance. First, FOSB knockdown specifically killed AqR cells relative to parental cells; second, FOSB knockdown abolished SCD levels in these cells; third, FOSB-silenced cells failed to acquire resistance to SCD inhibitors; last, FOSB silencing extended survival of mice treated with the SCD inhibitor. In conclusion, we showcase an unexpected finding of genetic and epigenetic silencing of a gene otherwise important and up-regulated in many human cancers. Because of the multiple direct and indirect beneficial effects of the SCD inhibitor, SCD expression may thus be used to stratify patients for SCD inhibitor clinical trials. Last, we demonstrate an evolutionarily conserved mechanism of acquired resistance to SCD inhibitor through drug-induced FOSB-mediated acute phase signaling response and target overexpression.

## MATERIALS AND METHODS

### Reagents

The following reagents were used: 5-Aza-2′-deoxycytidine, Chloroquine, Cyclohexemide, 4′,6-diamidino-2-phenylindole (DAPI), dimethyl sulfoxide (DMSO), hydrogen peroxide, corn oil, oleic acid, stearic acid ^13^C_18_ (all from Sigma-Aldrich), CAY10566 (Cayman Chemicals), MF438 (Millipore), Eeyarestatin (EMD Chemicals), Lapatanib (Selleck Chemicals), GSK2033 and GW9662 (from Tocris), methyl cellulose (Fisher Chemical), and d-luciferin (PerkinElmer). The VectaStain ABC kit, the DAB kit, and IsolectinB4 were from Vector Laboratories. Hematoxylin and eosin were from Richard-Allan Scientific.

### GBM tissue

Fresh frozen human tissue was obtained from the tissue repository at the University of Cincinnati under a UC institutional review board (IRB)–approved protocol.

### Cell culture

Human primary GBM lines were established from freshly resected tumors either in our laboratory under a University of Cincinnati IRB–approved protocol or obtained from our collaborators. Informed consent was taken from all subjects. Patient-derived glioma stem cell (GSC) lines G62 [epidermal growth factor receptor (EGFR) amplified and CDKN2A mutant], G68 (EGFR amplified, EGFRvIII positive, and CDKN2A mutant), and G82 (EGFR amplified, EGFRvIII positive, and CDKN2A mutant) were a gift from S. Lawler; HK281 (EGFR wt and CDKN2A wt) and GBM39 were a gift from F. Furnari; and TS600 (EGFR wt and CDKN2A mutant) and TS1156 (EGFR amplified and CDKN2A mutant) were a gift from C. Brennan. Cells were maintained as suspension cultures in ultralow attachment plates in GSC medium that contained glucose-free Dulbecco’s modified Eagle’s medium (DMEM)–F/12 supplemented with B27, EGF (10 ng/ml), basic fibroblast growth factor (10 ng/ml), and heparin (5 mg/ml). U87 (EGFR amplified, EGFRvIII positive, and CDKN2A mutant) and HEK 293T cells were purchased from American Type Culture Collection and were reauthenticated. These lines were maintained in DMEM high glucose supplemented with 10% fetal bovine serum (FBS). NHAs were purchased from Lonza Group Ltd. and immortalized with retroviral expression of Large T-antigen (pBabe-puro TcDNA, #14088, Addgene). NHAs were maintained in DMEM-F/12 supplemented with 10% FBS. Normal human melanocytes were a gift from Z. Abdel-Malek. Melanoma lines WM88, WM3331, WM852, and WM1232 were purchased from Rockland Immunochemicals, and A375, 451 LU, 1205 LU, and WM93B were gifts from R. Amaravadi. Melanoma lines were maintained in RPMI 1640 supplemented with 5% FBS. All cell lines were routinely checked for mycoplasma every other week using mycoplasma-specific PCR. Primary GSC lines and NHA as well as melanoma lines and melanocytes were analyzed by short tandem repeats (STR) analysis. For proliferation and viability analysis, both direct counting using trypan blue method and a fluorescence-based method (CellTiter-Fluor; Promega) were used.

### Plasmids T

The PLV-Nflag PTEN overexpression clone was provided by F. Furnari.

### Lentivirus preparation and production of stable shRNA/gRNA-expressing cell lines

Several independent shRNA clones were screened for each target gene, and the clones that exhibited maximal knockdown were used for the study. Most shRNA clones (in pLKO.1 plasmid) were from the Sigma Mission RNAi shRNA library. pLKO.1-puro scrambled (NT) shRNA (Sigma-Aldrich) was used as a negative control. shRNAs were prepared in 293T cells as before (*61*). NT and SCD gRNAs were purchased from genscript and prepared in 293T cells. Efficacy of knockdown/overexpression was assayed by WB or qRT-PCR. All shRNA/gRNA sequences are provided in table S5.

### RNA extraction and qRT-PCR

One microgram of RNA (RNeasy kit, Qiagen) was used for cDNA synthesis with oligo-dT primers and Multiscribe Reverse Transcriptase (Applied Biosystems). qRT-PCR was done using SYBR Green PCR Master Mix (Applied Biosystems) in a QuantStudio 6 Flex System (Applied Biosystems). Relative mRNA expression was calculated using the comparative Ct method after normalization to a loading control. Samples were run in triplicate. β-Actin was used as the loading control. Primer sequences are provided in table S5.

### Western blotting

WB analysis was carried out following standard methods as previously described (*62*). Each experiment was done at least two times. Antibodies were validated by using positive and negative control tissues and cells. Antibody information is provided in table S4. Protein expression was quantified using ImageJ.

### Electrophoretic mobility shift assay

Nuclear extracts from G82 and G82R cells were prepared by lysing cells in 10 mM Hepes (pH 7.9), 5 mM MgCl_2_, 0.25 M sucrose, and 0.1% NP-40 and centrifuging at 9800*g* for 10 min to remove the cytoplasmic extract. The pelleted nuclei were resuspended in 25 mM Hepes (pH 7.9), 20% glycerol, 1.5 mM MgCl_2_, 0.1 mM EDTA, and 700 mM NaCl; samples were then sonicated (65% for 5 cycles with 10-s pulses); and insoluble material was removed by centrifuging at 15,000*g* for 15 min. Total protein concentration of the nuclear extracts from each cell line was determined via Bradford assay. Two DNA probes containing the predicted FOS binding site or a mutated FOS site were created as previously described by annealing a 5′-IRDye 700 labeled oligo [Integrated DNA Technologies (IDT)] to the oligos listed (below) and filling in with Klenow (*63*). EMSAs were performed as previously described (*63*, *64*) with the following modifications: Nuclear extracts were added to binding reactions at a final concentration of 0.26 μg/μl of total protein, DNA probes were added to binding reactions last, and binding reactions were incubated at room temperature for 10 min before loading the gel. EMSAs were imaged using a Licor Biosciences Odyssey CLx scanner.

### Fluorescence in situ hybridization

FISH is a molecular cytogenetics technique whereby non-isotopically labeled DNA probes are hybridized to metaphase chromosomes and/or interphase nuclei. In this procedure, specimens are processed using a harvest technique, and CEPs (chromosome enumeration probes) and LSIs (locus specific identifiers) are hybridized to the metaphase chromosomes and/or interphase nuclei to identify numerical abnormalities and structural rearrangements. PTEN probe indicates 10q23, CEP 10 probe indicates chr 10 centromere, and SCD probe indicates RP11-34D15.

### Copy number variation analysis

CNV analysis was performed at the Cincinnati Children’s Hospital Medical Center (CCHMC) cytogenetics core facility. B allele frequency (BAF) is a measure of allelic intensity ratio. During deletion, BAF values cluster around 0 and 1 but are absent around 0.5. Log R ratio (LRR) is a normalized measure of total intensity signal. During deletion, the LRR value decreases below 0.

### Bisulfite pyrosequencing

For DNA measurement, a total of 500 ng of genomic DNA was incubated with sodium bisulfite and purified using the EZ DNA methylation-Gold kit. Pyrosequencing was carried out using Pyro Gold reagents with a PyroMark vacuum prep workstation and a PyroMark Q96 MD instrument. The generated pyrograms were automatically analyzed using Pyro Q-CpG methylation analysis software. Methylation control (100%) and 0% methylation control were used in validating all assays.

### PK studies

#### Animals

Adult female (201 to 250 g) jugular vein cannulated Sprague-Dawley rats (Charles River Laboratories) were used for this study. The experiments were conducted in strict accordance with the Institutional Animal Care and Use Committee (IACUC)–approved protocols of University of Cincinnati.

#### Construction of microdialysis probes

Concentric-style probes (210 μm outer diameter (O.D.) and 4.5-mm active length) were constructed with hollow fiber membrane with 13,000 Da molecular weight cutoff (Spectrum Laboratories, Rancho Dominguez, CA). The inlet tubing was PE-20 (0.38-mm inner diameter (I.D)., 1.09-mm O.D., 8-cm long; Becton Dickinson, Sparks, MD), and the outlet tubing was fused silica (75-μm I.D., 147-μm O.D., 5-cm long; Polymicro Technologies, Phoenix) within Tygon tubing (21-cm long, Thermo Fisher Scientific). All the probe components were glued to 26G hypodermic tubing (0.01 in I.D., 0.018 in O.D., 19-mm long) using epoxy. The average in vitro relative recovery of CAY10566 was 10%.

#### Microdialysis

Microdialysis probes were surgically implanted in jugular vein cannulated rats under anesthesia (ketamine/xylazine 70/6 mg/kg ip). Buprenorphine (Buprenex) was administered for perioperative analgesia. A probe was stereotaxically implanted in the striatum/caudate putamen (CPu) as per the stereotaxic atlas of Paxinos and Watson (antero-posterior, +1.2 mm; lateral, 3.1 mm; dorso-ventral, −7.8 mm, relative to bregma). Following implantation, the probes were continuously perfused with Dulbecco’s phosphate-buffered saline containing 1.2 mM CaCl_2_ and 5 mM glucose at a flow rate of 1 μl/min overnight. On the day of the experiment, the flow rate was increased to 2 μl/min, and the probes were allowed to equilibrate for an additional 2 hours before drug administration.

#### Drug administration and sample collection

CAY10566 (suspended in 0.5% Methocel in water with 0.2% Tween 80 at nine parts per one part DMSO stock) was administered at a final dose of 60 mg/kg via oral gavage. Blood and microdialysate samples were collected simultaneously for PK assessment at predose, 0.25, 0.5, 0.75, 1, 2, 3, 4, 6, 8, and 10 hours after administration followed by immediate centrifugation and separation of plasma from the blood samples. All the samples were stored at −80°C until analyzed.

#### Sample preparation

CAY10566 was extracted from the plasma using the protein precipitation technique. Calibration standards were prepared by spiking varying concentrations of CAY10566 in blank plasma or dialysate buffer. One hundred microliters of acetonitrile was added to 50 μl of plasma sample/standard for deproteinization. Following centrifugation (10,000 rpm for 10 min at 4°C), the supernatant was collected and analyzed using a validated LC/MS method. The dialysis samples, on the other hand, were injected directly into the chromatographic system.

#### PK data analysis

All the concentrations were time-averaged over the collection interval and were analyzed using a noncompartmental approach employing Phoenix WinNonlin 8.0 program (Certara). Key PK parameters estimated were the peak concentration (*C*_max_), time to reach *C*_max_(*t*_max_), area under the concentration-time curve between time 0 and the last observed concentration time point (AUC_0−*t*_), and the elimination *t*_1/2_. AUC_0−*t*_ values were calculated using the trapezoidal rule until the last concentration was measured. Results were expressed as means ± SD.

### Orthotopic xenograft

All animal procedures were carried out in accordance with the IACUC-approved protocol of CCHMC (Cincinnati, OH). Animals were monitored daily by veterinary services. For orthotopic implantation, 1 × 10^4^ primary human GBM cells (G82 or G82R), 1 × 10^6^ (HK281), or 1 × 10^5^ human GBM cell lines (U87) were stereotactically injected into the left striatum of NOD-SCID IL2Rg^null^ mice. CAY10566 was administered at 50 mg/kg via oral gavage, twice daily, with a drug holiday during the weekends. TMZ was administered at 100 mg/kg via oral gavage, once daily for 5 days. For in vivo bioluminescent imaging, luciferase expressing cells were established by infection with plenti-CMV-luc viral particles (a gift from S. Wells), and tumor growth was monitored using IVIS 200 system. Five minutes before bioluminescence imaging, mice were anesthetized and injected (intraperitoneally) with luciferin (150 mg/kg) and imaged using IVIS (Xenogen). All mice were euthanized following observation of lethargy and/or neurologic symptoms.

### Immunohistochemistry

IHC was performed as previously described (*62*). Mice were anesthetized and perfused intracardially with phosphate-buffered saline and 4% paraformaldehyde, and tumors were dissected and processed for paraffin embedding and sectioning. Antibody validation was done using multiple positive and negative control tissues and cells. Antibody information is provided in table S4.

### Tissue microarrays

TMAs were obtained from U.S. Biomax: GL806c (GBM), LV487b (liver), CO243b (colon), and ME481c (melanoma). In Fig. 1, for GBM, A1 and A2 through G9 and G10 represent tissues in duplicate for a total of 35 patients. For liver cancer, A1 and B1 through C8 and D8 represent tissues in duplicate for a total of 16 patients. For colon cancer, A1 and A2 through B5 and B6 represent tissues in duplicate for a total of six patients. SCD expression was quantified using ImageJ.

### Immunofluorescence microscopy

Confocal images were taken in a Nikon C2 confocal microscope. Antibody information is provided in table S4.

### Hypoxia

Cells were treated with 100 μM ^13^C_18_ stearate and maintained at hypoxia in a controlled atmosphere chamber (Don Whitley Scientific) with a gas mixture containing 0.1 to 1% O_2_, 5% (v/v) CO_2_, and 94% (v/v) N2 at 37°C for 24 hours.

### Lipidomics

Frozen cell pellets were extracted at 2 × 10^6^cells/ml using a Folch’s extraction as previously described (*65*). Supernatants (10 μl per injection) were analyzed on a Thermo Vanquish UHPLC coupled to a Thermo Q Exactive mass spectrometer in negative and positive ion modes (separate runs). Phases and chromatographic conditions for the 17-min negative method and 15-min positive method were previously described in detail (*65*). For both, the mass spectrometer operated in top 10 data dependent MS^2^ mode with normalized collision energy (NCE) of 30 eV [higher energy collisional dissociation (HCD)] and dynamic exclusion of 10 s. MS^1^ scans were acquired at a resolution of 70,000 across the *m/z* (mass/charge ratio) range of 125 to 1500. MS^2^ scans were acquired at a resolution of 17,500. Sheath and auxiliary gases (both N_2_) were set at 45 and 15 (unitless), respectively. Instrument stability and quality control were assessed via injection of technical replicates every six runs. Raw files were converted to mzXML using RawConverter. For the ^13^C stearate experiment, peak areas were extracted using Maven (Princeton University) and ^13^C-oleate assigned on the basis of intact mass, retention time matching with a commercial standard of oleic acid, and retention time discrimination against standards of constitutional isomers of oleic acid such as trans-vaccenic acid and elaidic acid. Untargeted lipid results were obtained using LipidSearch (Thermo Fisher Scientific) with precursor ion tolerance set to 5 ppm and product ion tolerance at 8 ppm. Annotated results were individually validated at the MS^1^ level using Maven; quantification of lipid peaks is based on precursor ion peak areas integrated in Maven. Total levels in each class were obtained for each replicate by summing all lipid peak areas. Precursor and fragment pairs mapping to more than one named lipid were discarded from calculations of total levels.

### RNA sequencing

One microgram of RNA was prepared and used for mRNA library preparation. Completed libraries were sequenced on an Illumina HiSeq2000 in Rapid Mode, generating 20 million or more high-quality 50 base long single end reads per sample.

#### RNA-seq data normalization and analyses

After alignment, the (BAM) files of RNA-seq data were analyzed using Avadis NGS software, version 1.3.0 against the RefSeq hg19 human genome annotation. DESeq normalization was performed on all samples. For DESeq normalization, the sequencing depth is estimated by the read count of the gene with the median read count ratio across all genes.

DESeq method via R script was performed on the filtered reads to identify the DEGs. The DESeq method used three functions (estimate size factors, estimate dispersions, and negative binomial test).The method is based on the negative binomial distribution, which allows for less restrictive variance parameter assumptions than does the Poisson distribution (*66*).

The false discovery rate (FDR) was calculated according to the Benjamini and Hochberg algorithm (*67*). Fold change (FC) of ±1.5 with an FDR of 0.05 was used as criteria for selection of DEGs. Heatmap and hierarchical cluster were constructed according to the normalized expression levels of mRNA. The eight samples (two parental lines in duplicate and two AqR lines in duplicate) were classified into two groups.

#### Functional classification and ontology of DEG

The functional classification of the DEGs identified was performed by IPA tool (www.ingenuity.com). The DEGs in the AqR cell line compared to WT were imported into the IPA knowledge base v6.3 for functional annotation that summarizes the DEGs associated with top biological functions and canonical pathways. Top canonical pathway comparison between AqR cells and parental cells was performed. The rankings were based on Fisher’s exact test, and high-ranking categories are displayed along in a decreasing order of significance from top. The cutoff for significance is *P* = 0.05.

#### Cluster analysis of gene expression profiles

Heatmaps were generated from hierarchical cluster analysis of the DEGs identified in the AqR cell compared to the WT samples. Hierarchical clustering was performed by Ward’s method using Euclidean distance metric.

### Assay for transposase-accessible chromatin using sequencing

ATAC-seq was performed as described in (*68*). Briefly, GSCs were spun down at 500*g* for 5 min at 4°C, and lysed in cold lysis buffer. After spinning down at 500*g* for 10 min at 4°C, nuclei were resuspended in transposition reaction mix and incubated for 30 min at 37°C. Immediately following transposition, DNA was purified using a QiagenMinElute PCR Purification Kit. Transposed DNA fragments were amplified and purified using the QiagenMinElute PCR Purification Kit. Libraries were generated using the barcoded primers in table S5.

#### ATAC-seq data analysis

FASTQ files, composed of 222,242,982 and 154,469,827 total sequencing reads for the parental and acquired resistance cell ATAC-seq experiments, respectively, were analyzed using the MARIO Pipeline (*69*). Briefly, the pipeline first runs QC on the FASTQC files containing the sequence reads using FastQQ (v0.11.2) (www.bioinformatics.babraham.ac.uk/projects/fastqc/). If FastQC detects adapter sequences, the pipeline runs the FASTQ files through Trim Galore (v0.4.2) (www.bioinformatics.babraham.ac.uk/projects/trim_galore/), a wrapper script that runs cutadapt (v1.8.1) (*70*) to remove the detected adapter sequence from the reads. The quality controlled reads were then aligned to the reference human genome (hg19/GRCh37) using bowtie2 (v2.3.4.1) (*71*). The aligned reads (in a .BAM format) were then sorted using samtools (v1.8.0) (*72*), and duplicate reads were removed using picard (v1.89) (https://broadinstitute.github.io/picard/). Last, peaks were called using MACS2 (v2.1.0) (https://github.com/taoliu/MACS), resulting in 54,415 and 60,339 ATAC-seq peaks for the parental and acquired resistance datasets, respectively.

The ATAC-seq experimental design consisted of replicate experiments of parental cells and acquired resistance cells. After independently analyzing the four datasets using the MARIO pipeline, we concluded that the replicates were highly similar (based on peak overlap). The .FASTQ files for the replicates were thus concatenated into a single set of reads for each of the parental and acquired resistance experiments, and alignment and peak calling were performed as described above.

To identify regions of differential chromatin accessibility between the parental and acquired resistance ATAC-seq datasets, we used MAnorm (*61*) with default parameter settings for read shift size (100), peak width (1000), and distance cutoff (500). To identify peaks unique to each cell types, we used a *P* value cutoff of 0.01 and a fold change cutoff of 1. These settings resulted in 9202 parental-specific peaks, 16,262 acquired resistance peaks, and 41,727 common peaks. Each peak set was then examined for enriched TF binding site motif instances using the HOMER suite of tools (*73*), modified to include the set of motifs contained in the Cis-BP database (*74*).

#### Prediction of FOS/FOSB binding sites within the SCD and FOS loci

Human FOS family (FOS, FOSB, FOSL1, and FOSL2) ChIP-seq experiments were identified in the Gene Expression Omnibus (GEO) Database through systematic searches. All resulting datasets were processed using the MARIO pipeline described above for the ATAC-seq experiments.

### Pooled shRNA screening

Cells were transduced with pooled shRNA viral particles (Custom shERWOOD-UltramiR lentiviral shRNA-mir pooled library; approximately 215 shRNAs targeting 39 genes in pZIP-mCMV-ZsGreen). Genomic DNA (gDNA) was extracted from the samples. The representation of each shRNA was detected by NGS. Individual shRNAs are amplified from gDNA with two rounds of PCR; the primary PCR amplifies the shRNA and the flanking region, and the secondary PCR uses nested primers to enrich for the primary PCR amplicons using modified primers adapted for NGS on an Illumina sequencer. NGS data were analyzed by calculating the nontarget (control) average and dividing that by the total number of reads.

### Data acquisition and analysis

Pan-cancer datasets in Fig. 3B were derived from Affymetrix Genome-Wide Human SNP Array 6.0. Data in Fig. 3C were derived from TCGA using GISTIC (Genomic Identification of Significant Targets in Cancer, version 2.0) module. For both Fig. 3 (B and C), SEMA, a graphical data mining software (http://semaomics.com/sema/), was used.

### Statistics and reproducibility

For all in vitro and ex vivo experiments, three to six technical replicates were used. Each experiment was repeated successfully two to three times as indicated in figure legends. For in vivo mouse orthotopic xenograft studies, four to six mice per group were used. Sample size was chosen with consideration to ensure adequate statistical power to detect prespecified effects. GraphPad Prism software was used to generate and analyze survival plot, and the R statistical program was used to generate box plots from TCGA data. *P* values were generated using a two-sided *t* test to calculate statistical significance with *P* < 0.05 representing a statistically significant difference. For Fig. 1 (L and M), a linear regression model was used to obtain statistical significance. The comparison was obtained from a linear model that included an indicator for the groups (sensitive/resistant) and the type and dose interaction. No statistical method was used to predetermine sample size. Kaplan-Meier analysis with log-rank post hoc test was used for survival studies. There was no need to exclude mice from analysis except the few that died during surgical transplantation of tumor cells. The number of indicated mice represents the total number of mice used and processed for each experiment. For orthotopic xenograft studies, mice were euthanized at the ethical end point when they failed to meet the predetermined CCHMC IACUC quality-of-life guidelines. No mice that completed in vivo studies were excluded from analyses. There are no limitations in reproducibility for experiments.

### Data storage

The information on the sequenced and partially processed RNA-seq and ATAC-seq datasets have been deposited to the NCBI’s GEO database. The GEO accession numbers to access the datasets are GSE131005 and GSE130638.

## SUPPLEMENTARY MATERIALS

Supplementary material for this article is available at http://advances.sciencemag.org/cgi/content/full/7/7/eabd7459/DC1

## Appendix

View/request a protocol for this paper from *Bio-protocol*.
